# Development of a novel human phage display-derived anti-LAG3 scFv antibody targeting CD8^+^ T lymphocyte exhaustion

**DOI:** 10.1186/s12896-019-0559-x

**Published:** 2019-10-17

**Authors:** Alessandro Ascione, Claudia Arenaccio, Alessandra Mallano, Michela Flego, Mara Gellini, Mauro Andreotti, Craig Fenwick, Giuseppe Pantaleo, Stefano Vella, Maurizio Federico

**Affiliations:** 1 National Center for Global Health, Istituto Superiore di Sanità (ISS), Viale Regina Elena 299, 00161 Rome, Italy; 2 Service of Immunology and Allergy, Lausanne University Hospital, University of Lausanne, Lausanne, Switzerland

**Keywords:** LAG3, Single-chain variable fragment, Phage display, Reconstituted scFv, CD8^+^ T lymphocytes, Lymphocyte exhaustion

## Abstract

**Background:**

Lymphocyte-activation gene (LAG)3 is a 498 aa transmembrane type I protein acting as an immune inhibitory receptor. It is expressed on activated lymphocytes, natural killer cells and plasmacytoid dendritic cells. In activated lymphocytes, LAG3 expression is involved in negative control of cell activation/proliferation to ensure modulation and control of immune responses. In view of its deregulated expression in tumor-infiltrating lymphocytes, LAG3, together with the additional immune checkpoint inhibitors CTLA4 and PD1, is considered a major target in order to reverse the immunosuppression typically mounting in oncologic diseases. Since many patients still fail to respond to current immune checkpoints-based therapies, the identification of new effective immune inhibitors is a priority in the ongoing fight against cancer.

**Results:**

We identified a novel human single-chain variable fragment (scFv) Ab against a conformational epitope of LAG3 by in vitro phage display technology using the recombinant antigen as a bait. This scFv (referred to as F7) was characterized in terms of binding specificity to both recombinant antigen and human LAG3-expressing cells. It was then rebuilt into an IgG format pre-optimized for clinical usage, and the resulting bivalent construct was shown to preserve its ability to bind LAG3 on human cells.

Next, we analyzed the activity of the anti-LAG3 scFvF7 using two different antigen-specific CD8^+^ T lymphocyte clones as target cells. We proved that the reconstituted anti-LAG3 F7 Ab efficiently binds the cell membrane of both cell clones after peptide-activation. Still more significantly, we observed a striking increase in the peptide-dependent cell activation upon Ab treatment as measured in terms of IFN-γ release by both ELISA and ELISPOT assays.

**Conclusions:**

Overall, the biotechnological strategy described herein represents a guiding development model for the search of novel useful immune checkpoint inhibitors. In addition, our functional data propose a novel candidate reagent for consideration as a cancer treatment.

## Background

Lymphocyte activate gene (LAG)3 (also known as CD223) is a transmembrane protein showing about 20% identity with CD4 (for a recent review, see [1]). Like CD4, LAG3 binds MHC Class II molecules, albeit at a distinct site and with a greater affinity [2, 3]. LAG3 is not expressed by resting T cells, although it is upregulated on activated T lymphocytes (both CD4^+^ and CD8^+^ subpopulations), B lymphocytes, natural killer cells, and plasmacytoid dendritic cells (DCs). Besides MHC Class II molecules, LAG3 has been found to bind Galectin-3 [4, 5], LSECtin [6] and, in central nervous system, α-synuclein [7]. LAG3 engagement leads to a negative regulation of T-cell activation, despite the LAG3 signal transduction mechanism being still largely unknown. A couple of unique, yet largely conserved domains, i.e., the EP [8] and KIEELE [9] motifs, seem important for the transmission of intracellular inhibitory signals. In addition, LAG3 shows a bidirectional signaling activity, as inferred by the modulation of DC activation/maturation observed upon interaction with LAG3 expressing lymphocytes [10, 11].

LAG3 has been identified as one of the major inhibitory receptors (IR) involved in the T lymphocyte exhaustion typically occurring in oncologic diseases [12]. For this reason, specific anti-LAG3 mAbs are currently under investigation in the attempt to reverse cancer-associated immunosuppression. Together with other IRs (i.e. CTLA4 and PD1) LAG3 is now considered a major target of the so called immune checkpoint blockade (ICB) antitumor strategy based on delivery of mAbs against IRs [13]. This therapeutic design has been positively adopted in the field of oncology, providing persistent clinical benefits for a significant number of patients with advanced cancer. Unfortunately, however, many patients fail to respond to ICB-based therapies, and the hyper-immune activation can associate with immune-related adverse events affecting several organs, including skin, gut, heart, lungs, and bone [14]. Therefore, nowadays the emphasis is on the evaluation of new immune checkpoint inhibitors and a combination thereof possibly increasing efficacy while also reducing toxicity [15], all resulting in a considerable interest in LAG3.

IRs maintain self-tolerance and modulate the duration and amplitude of physiological immune responses. It is now clear that tumors could co-opt certain regulatory pathways as a major mechanism of immune resistance, particularly in CD8^+^ T lymphocytes, and that this dysfunction could be reversed by anti-IRs antagonist mAbs [16]. Meanwhile, awareness is emerging that multiple IRs can be involved in lymphocyte exhaustion [17–19], so paving the way for more effective combinatorial approaches [20, 21]. A first confirmation of this hypothesis came from the fact that the combination of two IR blockers, namely anti-PD1 and anti-CTLA4 mAbs, resulted in a higher rate of response than that observed with single mAb treatments. In solid tumors, LAG3 is upregulated on tumor-infiltrating lymphocytes (TILs), while blockade of LAG3 can enhance anti-tumor T cell responses. On this basis, several reagents targeting LAG3 are currently being tested in various stages of preclinical and clinical development, thus attesting to the broad interest of this IR in the field.

Monoclonal Abs (mAbs) are molecules largely used in therapy, diagnostic and biotechnology. Among the various recombinant mAb formats, the single chain variable fragment (scFv) is one of the simplest and most versatile constructs that retains the antigen-binding activity [22]. This molecule is formed by the variable regions of both heavy and light chains joined by a peptide linker. While scFvs have the significant advantage that they can be expressed by a single open reading frame, their low stability and short pharmacokinetic may limit their applications. The scFv fusion with CH2 and CH3 domains of the Ig Fc region can overcome such drawbacks [23].

To avoid undesirable immune responses, human Abs represent the most suitable reagents for Ab-based therapeutic purposes. Human scFvs can be readily isolated through the well consolidated phage display technique. In this paper we describe the identification of a novel human anti-LAG3 scFv Ab recovered from a fully human naive phage library by means of an in vitro bio-panning strategy. Upon Fc-rebuilding, this Ab was shown to maintain its binding activity, while strongly increasing the activation of antigen-specific CD8^+^ T lymphocytes. We propose our anti-LAG3 Ab, in both scFv and reconstituted forms, as a candidate for novel biotechnology and clinic applications.

## Results

### Isolation of a novel human anti-LAG3 scFv ab by phage display technology

**Fig. 1:**
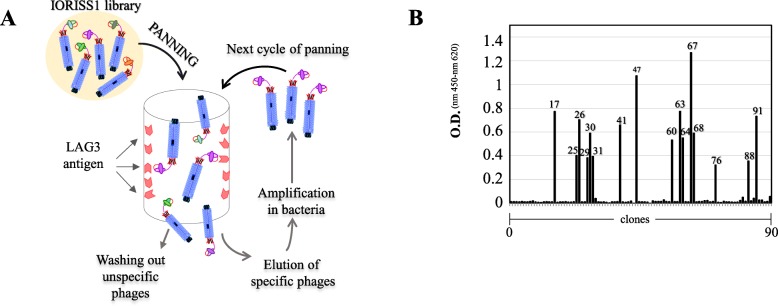
Isolation and screening of anti-LAG3 scFvs by phage display technology. **a** Scheme of the LAG3 affinity-based phage selection starting from the IORISS1 library. **b** Anti-LAG3 ELISA carried out on supernatants of 90 bacterial colonies isolated after five rounds of panning cycles. The plate was coated with 0.5 μg of antigen per microwell. The numbers identify each positive clone. O.D. nm 450 ref. 620: optical density

**Fig. 2:**
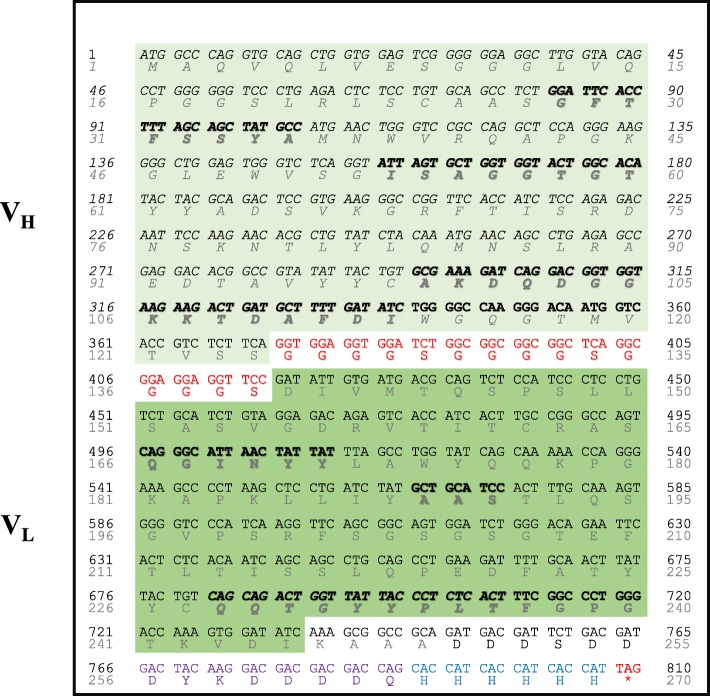
Nucleotide and amino acid sequences of scFvF7*.* The whole scFvF7 sequence, including tags, is reported. CDR1, CDR2 and CDR3 regions of both VH and VL chains are indicated in bold. The linker region is reported in red. The flag tag is indicated in violet, whereas the 6 × histidine region is in blue

Human recombinant scFvs against LAG3 were obtained by in vitro affinity enrichment process (bio-panning) as previously described [24], and depicted in Fig. 1a. In essence, an aliquot of phage-displayed scFvs representing the complexity of the naive IORISS1 library [25] was subjected to cycles of binding to recombinant LAG3 protein used as a bait and immobilized on immunotubes, followed by elution. Outputs after each round of selection were characterized by a progressive increase in phage titers (Table 1). Five rounds of selection were necessary to isolate a phage population detectable in anti-LAG3 ELISA. Soluble scFvs derived from single colonies were obtained after plating bacteria TG1 infected with the selected phages. Several of these scFvs were demonstrated to bind the recombinant LAG3 protein (Fig. 1b), whereas they were negative against an irrelevant antigen (GO, glucose oxidase) used as a negative control (data not shown). The scFv coding DNA was isolated from the positive clones, and the sequencing revealed a common genetic identity (Fig. 2).

**Table 1:**
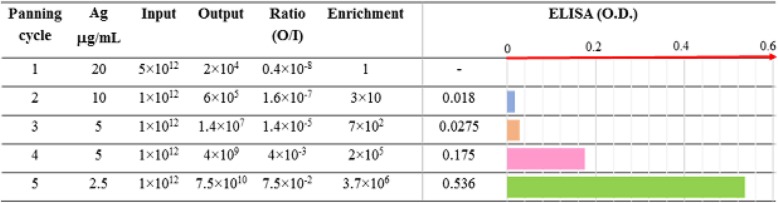
Selection of native IORISSI Ab phage library againts LAG3 recombinant protein^a^

^a^both Phage input (I) and output (O) were determined by titration in terms of colony forming units in infected *E. coli* TGI cells before and after each round of selection. Enrichment was calculated as ratio between outputs from each cycle and the output from the first one

**Fig. 3:**
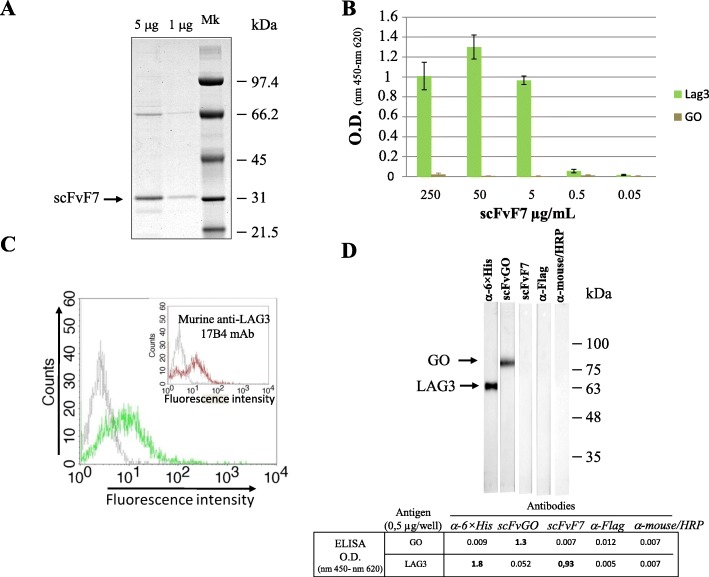
Characterization of the anti-LAG3 scFvF7. **a** SDS-PAGE analysis on 1 and 5 μg of scFvF7. Molecular markers in kilodaltons (kDa) are reported on the right. **b** ELISA for the detection of LAG3 antigen carried out with decreasing doses of purified scFvF7. GO: glucose oxidase . O.D.: optical density. The mean values ±SEM are shown as calculated from results of three independent assays. **c** FACS analysis carried out with scFvF7 on either unstimulated (grey line) or PHA-stimulated (green line) human CD4^+^ T lymphocytes. Inset: results from the same assay carried out with the murine anti-LAG3 17B4 mAb are reported. Four repeats of the experiment gave consistent results. **d** Binding analysis of scFvF7. 0.5 μg of both recombinant LAG3 and glucose oxidase (GO) proteins were loaded in each replicate well on a 12% SDS-PAGE under reducing condition and transferred to filter paper. Strips from the filter were then incubated with the indicted primary antibodies. Anti-6 his mAb was used as a positive control for LAG3 recombinant protein (which has a 6-histidines tag at its C terminal end). As additional negative controls, two strips were incubated respectively with the anti-Flag antibody and the HRP-conjugated anti-mouse antibody, in order to monitor eventual non-specific signals due to the secondary antibodies used to detect scFv antibodies. The ELISA shown below (performed with the same secondary Abs) is a check for the reactivity of the scFv-containing supernatants (scFvGO and scFvF7) used in the assay. Arrows indicate relevant signals. Molecular markers in kilodaltons (kDa) are reported on the right. A representative of three independent assays is shown

A representative clone, referred to as scFvF7, was produced in bacteria and purified by immobilized metal affinity *chromatography* using 6 × histidine-tag located at its C-terminus. SDS-PAGE analysis showed a single 31 kilodalton (kDa) band, at the expected size of the Ab fragment, with a negligible presence of both contaminate and degraded products (Fig. 3a).

The purified scFv was then tested for binding with recombinant LAG3 antigen in ELISA. The binding assay generated a specific dose-dependent association with LAG3 and a lack of binding against GO protein used as an irrelevant antigen (Fig. 3b).

Concerning the binding activity of scFvF7 on LAG3-expressing eukaryotic cells, flow cytometric analysis on CD4^+^ T lymphocytes carried out following a 3-day stimulation with phytohaemagglutinin (PHA) showed that scFvF7 binds native LAG3 with a comparable profile to that of a commercial murine anti-LAG3 Ab used as a control (Fig. 3c). Therefore, scFvF7 is capable of recognizing LAG3 antigen when it retains its native conformation, namely in ELISA on recombinant protein and in cytometry on live LAG3-expressing cells. Conversely, scFvF7 failed to react against its target in SDS-PAGE western blot assay (Fig. 3d), whose reducing/denaturing conditions stretch the amino acid structure of protein; this suggests a conformation-dependent epitope. An ELISA conducted on the heat-denatured LAG3 protein (by boiling) and a western blotting assay performed in non-reducing conditions strengthen this hypothesis (see Additional file 1: Figure S3D^II^).

### Engineering of divalent Fc-scFvF7 Ab

**Fig. 4:**
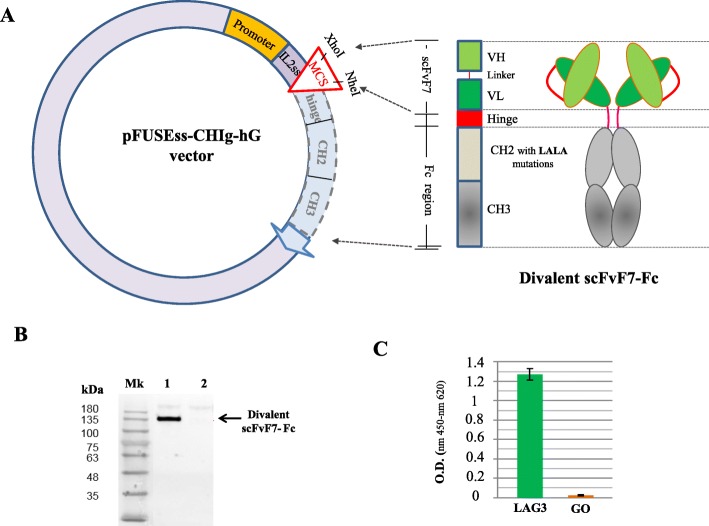
Construction and characterization of the divalent scFvF7-Fc Ab*.* **a** Scheme of the vector expressing the scFvF7-Fc open reading frame. Indicated are the locations of the hEF1α/HTLVI promoter, IL-2 signal sequences (IL-2 SS) acting as signal peptide, multiple cloninig site (MCS), and hinge. Domain map of the resulting protein product is also illustrated and, on the right, the overall structure of the divalent Ab. **b** Anti 6 × histidine western blot analysis carried on supernatants of CHO cells transfected with either the scFvF7-Fc expressing vector (lane 1), or transfected with the empty vector (lane 2). Molecular weight markers (Mk) are reported on the left. The results are representative of four independent assays. **c** *ELISA* performed with supernatants of CHO cells transfected with the vector expressing scFvF7-Fc. Either recombinant LAG3 or glucose oxidase (GO) were coated in the microwells (0.5 μg). The mean values ±SEM are shown as calculated from results of three independent assays

The use of single-domain Abs overcomes some limitations typical of tetrameric Abs, e.g., unspecific uptake, restricted access to tissues of interest. On the other hand, scFvs have been shown to have low in vivo stability as a consequence of their efficient blood clearance. Hence, to increase the expected in vivo stability of scFvF7 in view of possible clinical use, its fusion with CH2 and CH3 Fc domains was carried out. We generated a divalent scFvF7-Fc construct resulting in a recombinant Ab incorporating two scFvF7 molecules, each of them joined with CH2 and CH3 constant immunoglobulin domains. Similar to classic Abs, their spontaneous dimerization results in two anti-LAG3 binding sites on the same molecule, with the important advantage that the divalent molecule can be expressed by a single open reading frame (Fig. 4a). The presence of a hinge region between the scFv and the constant domains guarantees a flexibility similar to that of natural immunoglobulins. In addition, LALA mutations within the CH2 constant regions [26] would be particularly useful for a possible use in vivo since they are expected to lower the risk of unwanted Fc-dependent effects, e.g., Ab-dependent cell cytotoxicity. The scFvF7 open reading frame was cloned in frame with CH2 and CH3 Fc sequences in the context of the pFUSEss-CHIghGI vector which also expresses the human IgG1 secretory leader peptide sequence (Fig. 4a). The resulting molecular construct was transfected in CHO cells in order to verify the proper synthesis and secretion of the construct in an eukaryotic system. The western blot assay performed under non-reducing conditions on the supernatants of the transfected cells using anti-human Fc as detector showed a dominant signal compatible with the expected molecular weight (~ 140 kDa) of the reconstituted anti-LAG3 Ab (Fig. 4b). These supernatants efficiently and specifically reacted in LAG3 ELISA (Fig. 4c).

**Fig. 5:**
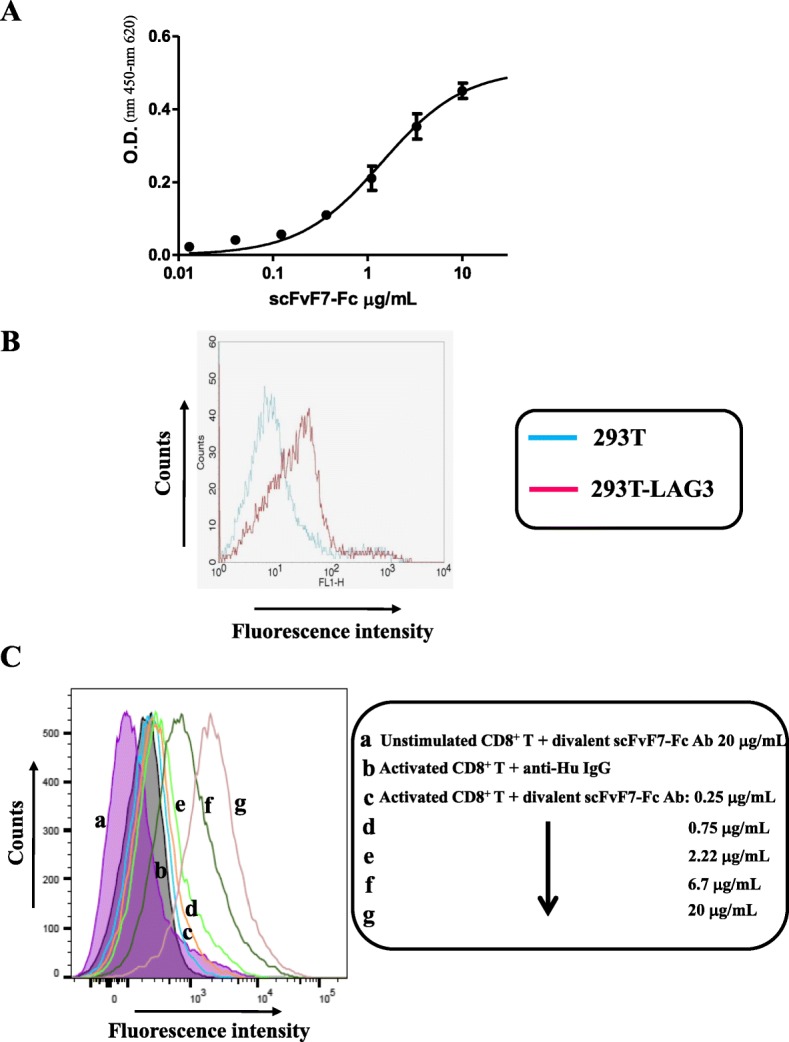
Binding properties of the divalent scFvF7-Fc Ab*.* **a** Binding curve of purified divalent scFvF7-Fc on recombinant LAG3. 0.1 μg of recombinant LAG3 was coated on ELISA microwells, and then different amounts of the reconstituted F7 Ab were incubated for one hour. The Ab binding was finally revealed through the incubation with an HRP-conjugated anti-human antibody. The mean values ±SEM are shown as calculated from quadruplicate wells from a representative of two independent experiments. O.D.: optical density (450 nm–620 nm). **b** FACS analysis on LAG3 overexpressing 293 T cells. Both parental and LAG3 engineered 293 T cells were incubated with the divalent scFvF7-Fc Ab, and then with FITC-conjugated anti-human IgGs. Data are shown from a representative of six independent experiments. **c** Dose-response binding of the reconstituted F7 Ab on cell membrane of activated human CD8^+^ T lymphocytes. PBMCs were isolated from the peripheral blood of healthy donors, and then the CD8^+^ T fraction was isolated by immunomagnetic selection. The cells were activated with PHA and, after six days, labeled with the indicated amounts of the divalent scFvF7-Fc Ab. As control, activated cells were labeled with the secondary Abs alone. As an additional control, unstimulated CD8^+^ T cells were labeled with the highest anti-LAG3 Ab concentration. The results shown are representative of two independent experiments

Next, the Fc-reconstituted F7 Ab was produced on a large scale by transient transfection in CHO cells, purified, and formulated in sterile PBS. The purified divalent Ab construct preserved its specificity as evidenced by both its reactivity in ELISA against recombinant LAG-3 (Fig. 5a), and its binding on human HEK 293 cells stably expressing LAG3 (Fig. 5b). Finally, FACS assays proved that the reconstituted F7 Ab binds human PHA-activated CD8^+^ T cells specifically, and in a concentration-dependent manner (Fig. 5c).

### Fc-reconstituted F7 Ab binds peptide-activated, antigen-specific CD8^+^ T lymphocytes

Besides CTLA4 and PD1, LAG3 is now considered a major target for the mAb-based cancer immunotherapy. ICBs are selected on the basis of their capacity to reverse the lymphocyte-dependent immune exhaustion, i.e., a typical signature affecting TILs infiltrating solid tumors, and particularly the CD8^+^ T sub-population [12]. In this context, the F7 Ab’s potential as a novel ICB candidate for new antitumor combination therapies relies on the assessment of its activity on antigen-specific CD8^+^ T lymphocytes. To this end, we carried out functional assays on a couple of antigen-specific CD8^+^ T cell clones, i.e., an HLA-B7 restricted clone specific for HIV-1 Nef, and an HLA-A.02-restricted clone specific for the human melanoma antigen Mart-1.

**Fig. 6:**
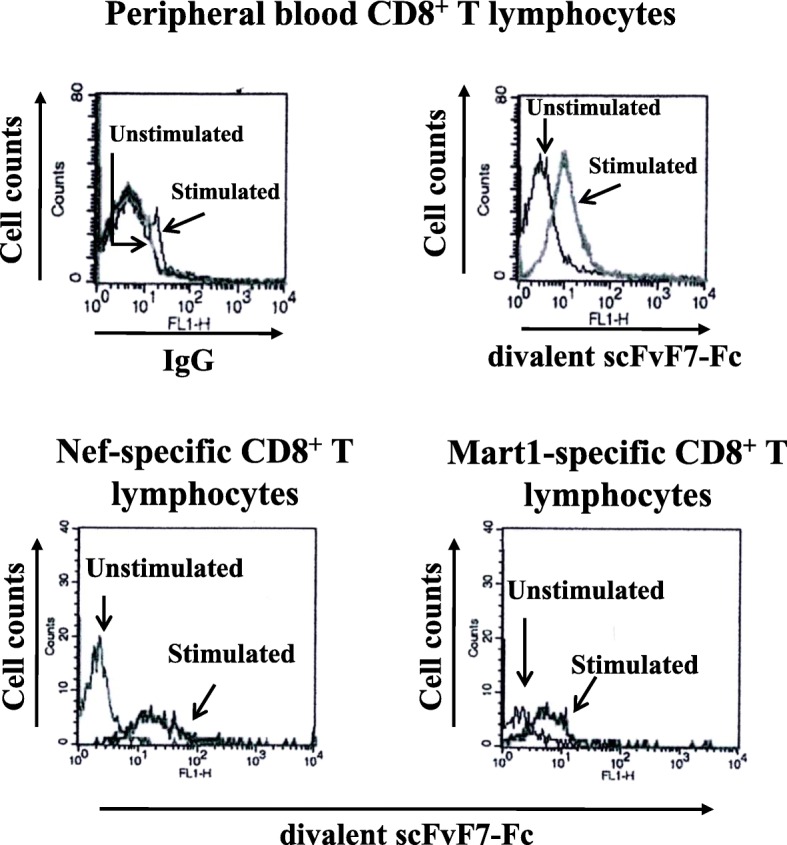
Binding of the divalent scFvF7-Fc Ab on antigen-specific CD8^+^ T lymphocytes. Both Nef-and Mart1-specific human CD8^+^ T lymphocytes were stimulated through co-cultivation with HLA-matched B-LCLs previously treated with either specific (stimulated) or mismatched (unstimulated) peptides. After o.n. co-cultivation, the co-cultures were labelled with the reconstituted F7 Ab, followed by incubation with FITC-conjugated anti-human IgGs. Then, cells were labeled with PE-conjugated anti-CD8 mAb. As control, PBMC-derived CD8^+^ T cells from either unstimulated or PHA-activated were labelled with either IgGs or the divalent scFvF7-Fc Ab (upper panels). In the bottom panels, histograms of FITC-related fluorescence profiles of PE-positive cells are shown. Results from a representative of four independent experiments are reported

Initially, we were interested in assessing the detection LAG3 expression with the reconstituted F7 Ab following the activation of the CD8^+^ T cell clones. The two clones were co-cultivated with HLA-matched B-lymphoblastoid cell lines (B-LCLs) previously treated with 100 ng/mL of each specific peptide. As a positive control, CD8^+^ T lymphocytes isolated from healthy donor peripheral blood mononuclear cells (PBMCs) were treated with PHA for three days. Stimulated cells were incubated with reconstituted F7 Ab and stained with a PE labeled anti-human IgG secondary Ab. As shown in Fig. 6, upon activation, both CD8^+^ T activated cell clones bound the reconstituted F7 Ab, a result which was consistent with the expected LAG3 upregulation.

These data validated the two CD8^+^ T cell clones as reliable substrates for functional assays devoted to assessing the activity of the divalent scFvF7.

### Increased IFN-γ production in antigen-specific CD8^+^ T lymphocytes treated with Fc-reconstituted scFvF7 Ab

**Fig. 7:**
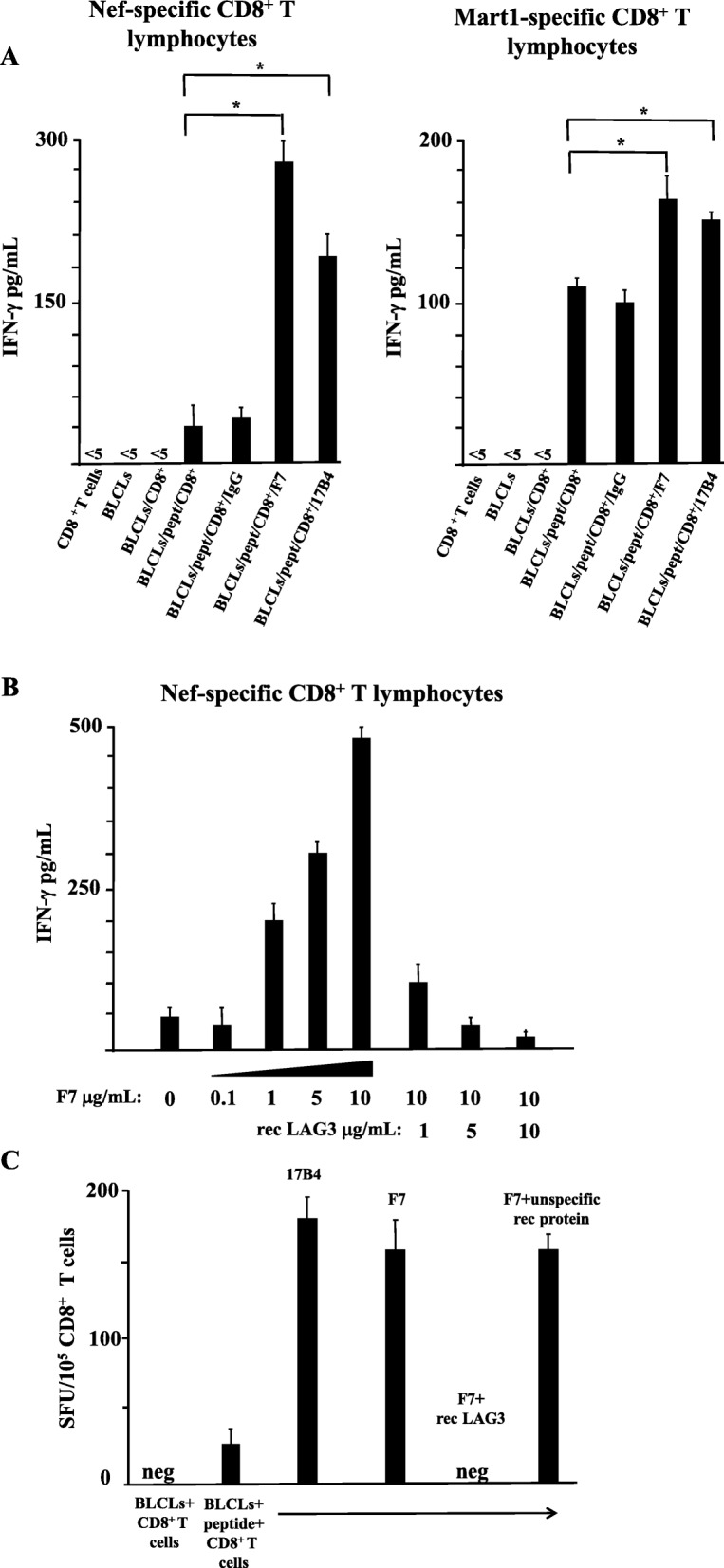
The treatment with the divalent scFvF7-Fc Ab increases the activation of peptide-stimulated antigen-specific CD8^+^ T lymphocytes*.* **a** Both Nef- and Mart1-specific CD8^+^ T lymphocytes were put in co-culture with HLA-matched B-LCLs previously treated with appropriate peptides, and in the presence of either IgGs, divalent scFvF7-Fc Ab, or the murine anti-LAG3 17B4 mAb. As control, both B-LCLs (either untreated or treated with appropriate peptides) and CD8^+^ T cells were cultivated either alone or in co-culture. After o.n. incubation, supernatants were harvested, clarified, and tested for IFN-γ contents. The mean values ±SEM are shown as calculated from triplicate wells from a representative of three independent experiments. **b** Dose-response effect of the divalent scFvF7-Fc Ab and inhibition by recombinant LAG3. Co-cultures comprising B-LCLs and Nef-specific CD8^+^ T lymphocytes were set up as reported for panel A, albeit in the presence of different amounts of the reconstituted F7 Ab. Conditions comprising the highest concentration of the divalent scFvF7 and increasing doses of recombinant LAG3 were also run. After o.n. cultivation, supernatants were tested for IFN-γ contents. The mean values ±SEM are shown as calculated from triplicate wells from a representative of two independent experiments. **c** Effects of the divalent scFvF7-Fc as sensed by IFN-γ ELISPOT assay. IFN-γ-specific CD8^+^ T cell response as detected by ELISPOT assay after o.n. co-cultures of Nef-specific CD8^+^ T cells with B-LCLs pre-incubated or not with the specific peptide. The co-cultures were run in the presence or absence of either the divalent scFvF7-Fc Ab or the murine anti-LAG3 17B4 mAb. The former co-cultures were carried also out also in the presence of either recombinant LAG3, or the same amounts of an unrelated recombinant protein (i.e., HIV-1 gp120). Results are expressed as spot-forming units (SFU)/10^5^ CD8^+^ T cells, and are representative of two experiments performed with triplicate wells

B-LCLs were treated with specific peptides for 3 h. The peptide loaded APCs were then co-cultured with the appropriate CD8^+^ T cell clones at a 1:1 cell ratio in the presence or absence of either an unspecific IgG control or anti-LAG3 Abs (either the reconstituted scFv or a murine mAb) in 100 μL of total volume. After an overnight (o.n.) incubation, supernatants were harvested, and IFN-γ production was evaluated by ELISA. The results reported in Fig. 7a (and Additional file 2: Figure S7A^II^, and Additional file 3: Figure S7A^III^) clearly demonstrate that the treatment with the Fc-reconstituted scFvF7 increased the IFN-γ release even more efficiently than the murine anti-LAG3 mAb validated for functional activity. The effect is more pronounced in the Nef-specific CD8^+^ T cells, this finding being consistent with the apparently higher level of LAG3 detected by the divalent scFvF7 Fc compared to that of Mart-1 specific CD8^+^ T lymphocytes (Fig. 6).

The specificity of the functional effect of scFvF7-Fc is supported by the data presented in Fig. 7b (and Additional file 4: Figure S7B^II^), where the divalent Ab was incubated with cells in the presence or absence of increasing amounts of recombinant LAG3 protein. The F7 Ab induced IFN-γ production in a concentration dependent manner that was effectively inhibited with the addition of the soluble LAG3 antigen. The specific effect of the divalent F7 Ab on the CD8^+^ T cell activation was further confirmed by IFN-γ ELISPOT assays (Fig. 7c and Additional file 5: Figure S7C^II^).

Taken together, our functional data indicate that the human divalent scFvF7-Fc Ab efficiently binds LAG3 on the surface of antigen-specific CD8^+^ T lymphocytes, thereby potently increasing their activation state. On the basis of its ability to counteract the LAG3 mediated inhibitory effect on antigen-activated CD8^+^ T lymphocytes, scFvF7-Fc can be proposed as a novel candidate for the treatment of pathologies characterized by LAG3-driven lymphocyte exhaustion.

## Discussion

Here we describe the experimental process aimed at the identification of novel anti-LAG3 human scFvs based on the previously described IORISS1 library [25]. This procedure allowed us to isolate the anti-LAG3 scFvF7. The combination of the results we obtained from western blot assay and ELISA are consistent with scFvF7 binding a conformational epitope of LAG3. Furthermore, functional assays carried out on antigen-specific CD8^+^ T lymphocytes demonstrated that the divalent Fc-fused scFvF7 biological activity is comparable to the functionally characterized murine anti-LAG3 17B4 mAb. Considering the human origin of scFvF7, these results are of particular relevance in view of possible in vivo uses of this scFv and derivatives thereof. In fact, monoclonal antibodies obtained with the classic hybridoma technology are extremely valuable instruments in many fields of application, but their use in the clinic is compromised by their xenogenic origin. Conversely, antibodies generated from human antibody libraries are entirely human, with the innate advantage of being only slightly or not at all immunogenic. Nevertheless, some drawbacks remain with the phage display-derived antibody fragments, such as their very short blood clearance. Therefore, the anti-LAG3 antibody here described was engineered in a recombinant ‘scFv-Fc’ format, in order to recover some important requirements for a potential drug compound, namely a structure with the typical avidity of natural antibodies (two binding sites), a prolonged half-life and a reduced ability to recall unwanted cell mediated responses [22, 23].

The interest in the identification and characterization of new ICBs is continually growing as witnessed by the varied clinical studies as well as by the quite recent publication of works describing the efforts to identify new anti-IR Abs. In particular, Everett et coll. Reported the isolation of novel anti-LAG3 Abs able to target both human and murine LAG3 by means of an additional engineered binding domain within the CH3 Fc region [27]. The in vitro activity of these Ab constructs resulted in an increased release of IL-2 from stimulated PBMCs. In addition, Sasso and colleagues described an Ab selection procedure based on binding phagemid particles on activated human lymphocytes to generate a collection of scFvs against different IRs including LAG3 [28]. Upon reconstitution into human IgG4 Fc, the respective biological activities were evaluated through cytometry-based lymphocyte proliferation assays and cytokine ELISA.

The biologic activity of scFvF7 was evaluated by means of a functional evaluation of CD8^+^ T lymphocytes specific for either viral or tumor antigens. We focused our interest on CD8^+^ T cells for the efficacy of scFvF7 as a new ICB candidate since CD8^+^ T-driven immune reactivation is expected to have a significant therapeutic effect to counteract the exhaustion of this lymphocyte subpopulation in cancer [29] and even chronic infectious diseases [30].

Overall, it is now clear that the broad success of ICB-based immunotherapies will depend on the combination of different treatment options. In a rapidly evolving research landscape, the generation of a repertoire of different immune-modulatory Ab-based constructs could play an essential role in identifying novel therapeutic agents and LAG3 certainly represents one of the most appealing emerging targets. The great interest in LAG3 has been fueled by its sustained expression in dysfunctional T-cells associated with many human tumors [31–33]. In addition, there is strong evidence that LAG3 upregulation could mediate an escape mechanism from PD1 therapy, whose acquired resistance might be overcome by the combined treatment with anti-LAG3 ICBs [34–37]. Another more recent study, for its part, highlights how LAG-3 blockade could result in a superior T cell activation compared to other inhibitory pathways, including PD1/PDL-1 axis [38]. Furthermore, LAG3, together with TIGIT and Tim-3, has been predicted to have a better safety profile in the clinic compared to CTLA4 and PD1 combinations [1, 15]. Also, LAG3 blockade inhibits the suppressive activity of T_reg_ cells both in vitro and in vivo in a model of autoimmune pulmonary vasculitis [39], thus raising the intriguing hypothesis that a similar beneficial effect may be obtained in the tumor microenvironment. For all these reasons, LAG3-specific ICBs are currently being evaluated in phase I and phase II trials in a variety of solid and hematological malignancies either as monotherapy or in combination with anti-PD1 antibodies.

## Conclusions

The increasing number of Abs against emerging regulatory molecules is expected to play a key role in promoting systematic analyses for the definition of robust biomarkers useful in predicting therapeutic activity and/or toxicity, as well as in designing patient-tailored precision medicine combination treatments. In the present study we describe the isolation and functional activity of a new anti-LAG3 recombinant Ab. Whereas further characterization studies are needed to support the potential of scFvF7 as a clinical candidate for the treatment of disease conditions in combination with other ICBs, data presented supports this agent as a valid scaffold for the development of novel anti-LAG3-based interventions.

Finally, in addition to the human nature of the scFvF7, making it a suitable reagent for clinical application, the flexibility of the described biotechnological platform allows an easy exploration of alternative therapeutic constructs, including bispecific or bifunctional Abs. These novel Abs could also have enhanced functional activity by substituting one antigen-binding portion with other ligands/cytokines and promoting the pairing of the heterodimers through insertion of specific mutations (e.g. Knob/Hole) [40] in constant regions.

## Methods

### Ab phage library

The IORISS1 naive human Ab library [24] consists in a large array (i.e., more than 10^9^ Ab combinations) of scFv polypeptides displayed on the surface of M13 phage. It was constructed by recovering light and heavy Ab chains from a group of healthy donors, then randomly joining them by means of an appropriate peptide linker, and cloning the resulting scFvs into a phagemic vector as previously described [25]. It is noteworthy that the scFv inserts have been cloned upstream of two tag sequences, Flag tag and 6-his tag, respectively suitable to detect scFv in immunochemical assays and to purify them by nichel chromatography.

### Biopanning strategy for selection LAG3 specific scFvs

LAG3-specific scFv Abs were selected from IORISS1 phage library as described elsewhere [25]. Briefly, a number of phages (~ 10^13^ colony forming units, *cfu*), proper to represent the complexity of the library, was placed in an immunotube (Nunc Maxisorp) previously coated o.n. at room temperature (r.t.) with 4 mL of phosphate buffered saline (PBS) containing the recombinant LAG3 antigen (Acrobiosystem) at a concentration of 5 μg/mL. After 2 h of incubation, the specific phages were detached by an elution carried out with a solution of 100 mM triethylamine (1 mL), ten minutes later neutralized with 0.5 mL of 1 M Tris–HCl pH 7.4. Afterwards, the phages were amplified in TG1 *E. coli* bacteria [supE hsd15 thi 1(lacproAB) F′(traD36 proAB+ lacIqlacZ1M15)], as previously reported [25]; in detail, phages were used to infect TG1 bacteria in exponential growth phase, and the latter distributed on a plate with solid selective medium consisting of 2xTY (Sigma), 2% glucose and ampicillin (100 μg/mL). The day after, 50 mL of liquid selective medium was inoculated with the bacteria grown on the selective plate in order to obtain a 600 nm optical density (O.D.) of 0.05–0.1; when the culture reached the O.D. of 0.4–0.5, the bacteria were re-infected with the M13 K07 helper phage in a ratio phages/bacteria of about 20: 1, and incubated at 30 °C o.n.. Finally, the phages released into the supernatant were precipitated by means of PEG (PolyEthylene Glycol) 6000 before being used for a new cycle of selection on the LAG3 antigen.

An aliquot of TG1 bacteria infected with the phages from each panning cycle were also induced with isopropyl β-dithiogalactopyranoside (IPTG, Sigma) (final concentration > 1 mM) and used to perform a policlonal ELISA in order to monitor the enrichment of the LAG3-specific population (Table 1). In addition, bacteria infected with phages from the last panning was properly diluted and spread on selective plates in order to obtain single bacterial clones to be tested for their ability to secrete LAG3-specific scFvs.

For ELISA monoclonal screening, one hundred individual clones were inoculated in 96-well plates (Nunc) in a selective medium with 0.1% glucose; after 2 h of growth at 37 °C, clones were induced as above described and incubated for additional 12 h at 30 °C with shacking. Therefore, the scFv-containing supernatants were recovered, spun down (1800×*g* for 10 min) and used as primary antibodies against LAG3 protein.

### DNA characterization and sequences

Plasmid DNAs from individual bacterial colonies were amplified and analyzed by restriction enzyme digestion using *Nco* I and *Not* I (i.e., the restriction sites used to clone the antibody fragments in IORISS1 library) to verify the presence of the scFv-encoding inserts. Afterwards, the scFv ORFs from the positive clones were sequenced with an automated DNA sequencer (Eurofins Genomics) using the following primers: scFv forward: 5′-atgaaacaaagcactattgcact-3′; scFv reverse: 5′-ttgatattcacaaacgaatgg-3′. The sequences were finally analyzed using the IMGT database.

### ELISA on recombinant LAG3 protein

For screening procedure, ninety-six-well ELISA plates were coated with 50 μL/well of 10 μg/mL of recombinant LAG3 in PBS at 4 °C. The following day, a blocking solution consisting of 2% non-fat milk in PBS (MPBS) was added, and after 2 h the plates were washed with PBS containing 0.1% Tween 20 (TPBS). Plates were incubated for 2 h at r.t. with 50 μL of supernatants containing soluble scFv Abs, anti-flag M2 Ab (2.5 μg/mL, Sigma), and HRP- conjugated anti-mouse Ab (5 μg/mL, Dako). All Abs were resuspended in 2% MPBS. The reaction was visualized with 3,3′-5,5′-tetramethylbenzidine (BM blue, POD substrate, Roche), and stopped by adding 50 μL of 1 M sulfidric acid. The reaction was detected by an ELISA reader (Model 680 microplate reader, BioRad), and results expressed in terms of absorbance (A) = A_450 nm_-A_620 nm_.

The same ELISA procedure described above was performed to verify the reactivity of the scFvs-containing bacterial supernatants that were used for the Western blot assays.

The coating step for the assays to test the transfected CHO-derived supernatants was performed, as previously with 0.5 μg of recombinant LAG3/well, while for the dose-response binding analysis of the purified Fc-reconstituted scFv, the antigen was lowered to 0.1 μg /well. In both cases, the reconstituted Ab was incubated for one hour at r.t., followed by the incubation with a HRP-conjugated anti-human Ab (Thermo Fisher Scientific). The remaining procedures were performed as above described.

### Soluble scFv purification

For the production of soluble scFvs, specifc phage-TG1 *E. coli* infected cells were cultured at 37 °C in 2 × TY containing 100 μg/mL ampicillin and 0.1% glucose up to 0.5 O.D. After induction of Ab expression by adding IPTG 1 mM, cells were incubated o.n. at 30 °C. Then, the bacterial cultures were centrifuged, and scFv containing supernatants collected. ScFv Abs were precipitated with ammonium sulfate, and then dialyzed in PBS. 6 × His-tagged scFv Abs were purified by immobilized metal affinity chromatography using Ni_2+_-nitriloacetic acid agarose (Qiagen). ScFv fragments were eluted with 250 mM imidazole in PBS, dialyzed, aliquoted, and stored at − 80 °C.

### Eukaryotic cell cultures

PBMCs were obtained by Ficoll–Hypaque density gradient centrifugation from heparinized blood samples of healthy donors. Purified T cells were obtained by PBMC negative selection using a kit for human T cell selection (Miltenyi) according to the manufacturer’s protocol, and cultivated in RPMI medium supplemented with 10% of heat-inactivated fetal calf serum (FCS)(HyClone). Both CHO and HEK293T cells were cultivated in DMEM plus 10% FCS. Both HLA-A02 and HLA–B7 B-LCLs were grown in RPMI medium supplemented with 10% FCS. B-LCLs were generated by in vitro EBV infection of ex vivo B lymphocyte cultures. Both isolation and expansion of CD8^+^ T cell clones specific for Mart (melanoma-associated recognized by T cells)-1 and HIV-1 Nef have been previously described [41, 42]. Mart1-specific CD8^+^ T cells recognize the HLA-A.02-restricted AAGIGILTV_27–35_ peptide sequence, whereas Nef-specific CD8^+^ T cells recognize the HLA-B7-restricted TPGPGVRYPL_128–137_ peptide. Both antigen-specific CD8^+^ T-cell clones were cultivated in RPMI plus 10% AB human serum (Gibco), and regularly monitored for their specificity.

### Flow cytometry assays on human T cells

T cells isolated from PBMCs were seeded at the concentration of 2 × 10^6^/mL, and activated with 5 μg/mL of PHA. During the screening phase, scFvF7 was preliminarily tested on TCD4 cells at a one shot concentration (50 μg/mL), just to have a ‘yes/no’ response about its ability to recognize the antigen on the cell surface. About 1 × 10^6^ cells were resuspended in PBS containing primary scFv antibody and incubated for 1 h at r.t.. After washing, cells were resuspended for 30 min at 4 °C in PBS containing 25 μg/mL of an anti-FLAG M2 mouse antibody (Sigma) and then incubated for another 30 min at 4 °C with 6 μg/mL of an FITC-goat anti-mouse IgG (Pierce, IL, USA). An irrelevant antibody directed to an irrelevant antigen (glucose oxidase, GO) was used as negative control. After staining, the cell samples were washed, maintained at 4 °C and immediately analyzed by FACScan (Becton-Dickinson, NJ, USA) equipped with 15 mW, 488-nm, argon laser. Fluorescence compensation was determined using samples stained with anti-glucose oxidase scFv and goat FITC-conjugated anti-mouse secondary antibody.

For the cytofluorimetric analysis on HEK293 T cells (both LAG3-negative parental and the derivative LAG3 expressing cell lines, kindly provided by Pantaleo’s team, Lausanne), 2.5 × 10^5^ cells were incubated with Fc-reconstituted scFvF7 Ab (10 μg/mL) for 1 h at r.t., and after washing with an goat FITC-conjugated anti-human IgG1 (Pierce) for 30 min at 4 °C.

Similarly, both PBMC-derived T cells and antigen-specific CD8^+^ T lymphocytes (1 × 10^6^ cells/test) were incubated with Fc-reconstituted anti-LAG3 scFv, at different concentrations as specified in each experimental assay (comprised between 0.1 and 20 μg/mL) for 1 h at r.t.; the murine 17B4 anti-LAG3 mAb (EnzoLab, 10 μg/mL) was used as control. Then, cells were washed and incubated with FITC-conjugated secondary either anti-human (for detecting Fc-reconstituted scFvF7) or anti-mouse (for detecting mouse 17B4 mAb) goat IgG1 (Pierce). In all cases, stained cells were analyzed on FACscan platform (BD Biosciences).

### Western blotting

For the analysis of the scFv binding in Western blot assay, 0.5 μg of both recombinant LAG3 and, as control, glucose oxidase (GO), were loaded in multiple wells, run on 12% SDS-PAGE (under reducing or non-reducing conditions, as specified in each assay) and transferred to nitrocellulose membrane. Separate filter strips were then incubated for 2 h with bacterial supernatants from the anti-LAG3 clone scFvF7 and, as control, from a previously characterized anti-GO clone (scFvGO) [24], both of them previously tested in ELISA to verify their reactivities. Afterwards, the strips were washed and incubated again with 5 μg/mL anti-flag M2 mouse antibody (Sigma) in 2% MPBS. After an additional incubation for 1 h at r.t. in the presence of 5 μg/mL of a goat anti-mouse antibody HRP-conjugated (Dako), the reaction was developed and visualized using a chemiluminescence reagent (NEN Life Science Products). A mouse anti-6 × His mAb (Serotec, 1 μg/mL diluted in 2%MPBS) was used as positive control to detect the recombinant LAG3 protein (which is provided with a 6 × His-tag at its C-terminus).

For monitoring transfection efficiency of CHO cells with the vector encoding the Fc-reconstituted anti-LAG3 antibody, supernatants from transfected cells (50 μL/lane) were separated by 10% SDS-PAGE, and then transferred to nitrocellulose membrane. After blocking with 5% MPBS, the membranes were washed and incubated for 1 h at r.t. with HRP-conjugated, goat anti-human IgG (1:2000 dilution, (Thermo Fisher Scientific)) and developed as above described.

### Genetic construction and production of the divalent anti-LAG3 scFvF7-Fc Ab

The anti-LAG3 scFv coding sequence was amplified from IORISS1 phagemide vector [24] by PCR with tailored primers. The amplified fragment was then digested with *Xho* I and *Eco* RI enzymes, gel purified, and ligated in frame to the *Xho* I*/Eco* RI double digeted pFUSEss-CHIg-HG1 vector (Invivogen) expressing the sequences of both human Fc CH2, including LALA mutations [26], and CH3 Fc domains. The resulting construct was transfected into CHO cells for pilot production of bivalent scFvF7-Fc Ab. Briefly, 10^6^ cells were transfected with 1 μg of plasmid DNA per well in a six-well tissue culture plate using Lipofectamine Plus reagent (Life Technologies) according to the manufacturer’s instructions. Supernatants were collected 48 h later to evaluate Ab production through both western blot analysis and ELISA.

### Large-scale production of the divalent scFvF7-Fc Ab

Divalent scFvF7-Fc Ab was produced through transient transfection of CHO DG44 cells followed by incubation for 6 days in ProCHO5 serum free medium (Lonza). The scFvF7-Fc Ab was purified from the cell medium using a protein A column (Thermo Fisher) with Abs eluted with 100 mM glycine buffer pH 3.0 into a 1 M Tris-HCl eluate at pH 8.0. Abs were dialyzed twice against PBS, concentrated using a JumboSep centrifuge filter with 3 kDa molecular weight cut-off (Pall Laboratories), and sterile filtered with Millex GP 0.22 μm pore size (Millipore). Prior to evaluation of scFvF7-Fc in the in vitro functional recovery assays, Abs were tested with the FDA-licensed Endosafe-PTS kit (Charles River Laboratory) which showed < 5 EU of endotoxins per mg of protein.

### IFN-γ ELISA and ELISPOT assays

CD8^+^ T cells were pre-incubated with different concentrations of the reconstituted anti-LAG3 scFv for 2 h, and then co-cultured at a 2:1 ratio with HLA-matched B-LCLs previously pulsed with 100 ng/mL of the appropriate peptide. The co-cultures were incubated o.n., and finally the supernatants were clarified and assayed for IFN-γ content by ELISA (Immunological Sciences). For ELISPOT assays, the co-cultures were carried out for 16 h in ELISPOT microwells previously coated with a mAb against human IFN-γ (clone D1K, Mabtech). Afterwards, the cells were removed, and a biotinylated Ab against human IFN-γ added, followed by the addition of a streptavidin-alkaline phosphatase. The plate was then developed using BCIP/NBT substrate (Sigma). Spot-forming cells were analyzed and counted using an ELISPOT reader (Amplimedical Bioline A-EL-VIS GmbH).

### Statistical analysis

Statistical analyses were performed by using GraphPad Prism version 5.01 software. Data are expressed as means ± standard error of the mean (SEM). The student’s *t* test was used to determine significance. A *p* value of < 0.05 was considered statistically significant.

## Supplementary information

**Additional file 1: Figure S3D**^**II**^**.** Characterization of the anti-LAG3 scFvF7 (II). A. ELISA conducted in parallel on intact recombinant LAG3 protein and heat-denatured LAG3 protein (by boiling for 5 min). The plate was coated with 0.5 μg of antigen per microwell (GO, intact recombinant LAG3 and heat-stressed recombinant LAG3). After blocking step (2% MPBS for two hours at r.t.) wells were incubated for 2 h at r.t. with 50 μL of scFvF7 (25 μg/mL) together with anti-flag M2 Ab (2.5 μg/mL, Sigma) and HRP- conjugated anti-mouse Ab (5 μg/mL, Dako). A mouse anti-6 his mAb (which recognizes the 6-histidines tag at C terminal end of LAG3 protein)(Serotec) and commercial mouse anti-LAG3 mAb 17B4 (that recognizes the 30 aa extra-loop of the first N-terminal D1 domain of human LAG3)(EnzoLab) were used as positive controls. All Abs were resuspended in 2% MPBS. O.D.: optical density. B. Western blotting assay with LAG3 under reducing and non-reducing conditions. 0.5 μg of glucose oxidase (GO) and of recombinant LAG3 proteins (the latter under three different conditions, namely: -DTT (reducing agent)/−boiling, −DTT/+boiling 5 min, +DTT/+boiling 5 min) were loaded as specified in replicate wells on a 12% SDS-PAGE and transferred to filter paper. Portions from the filter were then incubated with the indicated primary antibodies. An Anti-6 his mAb was used as a positive control for LAG3 recombinant protein (which has a 6-histidines tag at its C terminal end). Arrows indicate relevant signals. The different molecular weights observed for LAG3 are obviously attributable to the impact of the different experimental conditions (non-reducing and reducing) on the SDS-PAGE separation. Molecular markers in kilodaltons (kDa) are reported on the right. The reactivity of the supernatants (scFvGO and scFvF7) used was previously checked in ELISA (bottom). (PPTX 125 kb)

**Additional file 2: Figure S7A**^**II**^**.** The treatment with the divalent scFvF7-Fc Ab increases the activation of peptide-stimulated Nef-specific CD8^+^ T lymphocytes in terms of IFN-γ secretion (Exp. I and Exp. III). For details see Legend of Fig. 7a. (PPTX 40 kb)

**Additional file 3: Figure S7A**^**III**^**.** The treatment with the divalent scFvF7-Fc Ab increases the activation of peptide-stimulated Mart1-specific CD8^+^ T lymphocytes in terms of IFN-γ secretion (Exp. II and Exp. III). For details see Legend of Fig. 7a. (PPTX 41 kb)

**Additional file 4: Figure S7B**^**II**^**.** Dose-response effect of the divalent scFvF7-Fc Ab and inhibition by recombinant LAG3 (Exp. II). For details see Legend of Fig. 7b. (PPTX 36 kb)

**Additional file 5: Figure S7C**^**II**^**.** Effects of the divalent scFvF7-Fc as sensed by IFN-γ ELISPOT assay (Exp. II). For details see Legend of Figure 7c. (PPTX 36 kb)

## Data Availability

The datasets used and/or analyzed during the current study are available from the corresponding author on reasonable request.

## Abbreviations

B-LCLs: B-lymphoblastoid cell lines
DCs: Dendritic cells
ELISA: *Enzyme-linked immunosorbent assay*
*ELISPOT*: Enzyme-linked immunospot
Fc: Fragment crystallizable
FCS: Fetal calf serum
GO: Glucose oxidase
HLA: Human leukocyte antigen
ICB: Immune checkpoint blockers
IFN: Interferon
Ig: Immunoglobulin
IPTG: Isopropyl β-dithiogalactopyranoside
IR: Inhibitory receptor
kDa: Kilodaltons
LAG3: Lymphocyte activate gene-3
mAb: Monoclonal antibody
O.D.: Optical density
o.n.: Overnight
PBMCs: Peripheral blood mononuclear cells
PBS: Phosphate buffered saline
PHA: Phytohaemagglutinin
r.t.: Room temperature
scFv: Single-chain variable fragment
SE: Standard error
TILs: Tumor-infiltrating lymphocytes
