# Supplementary material for: Development of a novel human phage display-derived anti-LAG3 scFv antibody targeting CD8+ T lymphocyte exhaustion
Source: BMC Biotechnol. 2019 Oct 17;19:67. doi: 10.1186/s12896-019-0559-x (PMC6798348; doi:10.1186/s12896-019-0559-x)
Supplement: Supplementary file 1 — Additional file 1: Figure S3DII. Characterization of the anti-LAG3 scFvF7 (II). A. ELISA conducted in parallel on intact recombinant LAG3 protein and heat-denatured LAG3 protein (by boiling for 5 min). The plate was coated with 0.5 μg of antigen per microwell (GO, intact recombinant LAG3 and heat-stressed recombinant LAG3). After blocking step (2% MPBS for two hours at r.t.) wells were incubated for 2 h at r.t. with 50 μL of scFvF7 (25 μg/mL) together with anti-flag M2 Ab (2.5 μg/mL, Sigma) and HRP- conjugated anti-mouse Ab (5 μg/mL, Dako). A mouse anti-6 his mAb (which recognizes the 6-histidines tag at C terminal end of LAG3 protein)(Serotec) and commercial mouse anti-LAG3 mAb 17B4 (that recognizes the 30 aa extra-loop of the first N-terminal D1 domain of human LAG3)(EnzoLab) were used as positive controls. All Abs were resuspended in 2% MPBS. O.D.: optical density. B. Western blotting assay with LAG3 under reducing and non-reducing conditions. 0.5 μg of glucose oxidase (GO) and of recombinant LAG3 proteins (the latter under three different conditions, namely: -DTT (reducing agent)/−boiling, −DTT/+boiling 5 min, +DTT/+boiling 5 min) were loaded as specified in replicate wells on a 12% SDS-PAGE and transferred to filter paper. Portions from the filter were then incubated with the indicated primary antibodies. An Anti-6 his mAb was used as a positive control for LAG3 recombinant protein (which has a 6-histidines tag at its C terminal end). Arrows indicate relevant signals. The different molecular weights observed for LAG3 are obviously attributable to the impact of the different experimental conditions (non-reducing and reducing) on the SDS-PAGE separation. Molecular markers in kilodaltons (kDa) are reported on the right. The reactivity of the supernatants (scFvGO and scFvF7) used was previously checked in ELISA (bottom). (PPTX 125 kb) [file 12896_2019_559_MOESM1_ESM.pptx]

## Slide 1
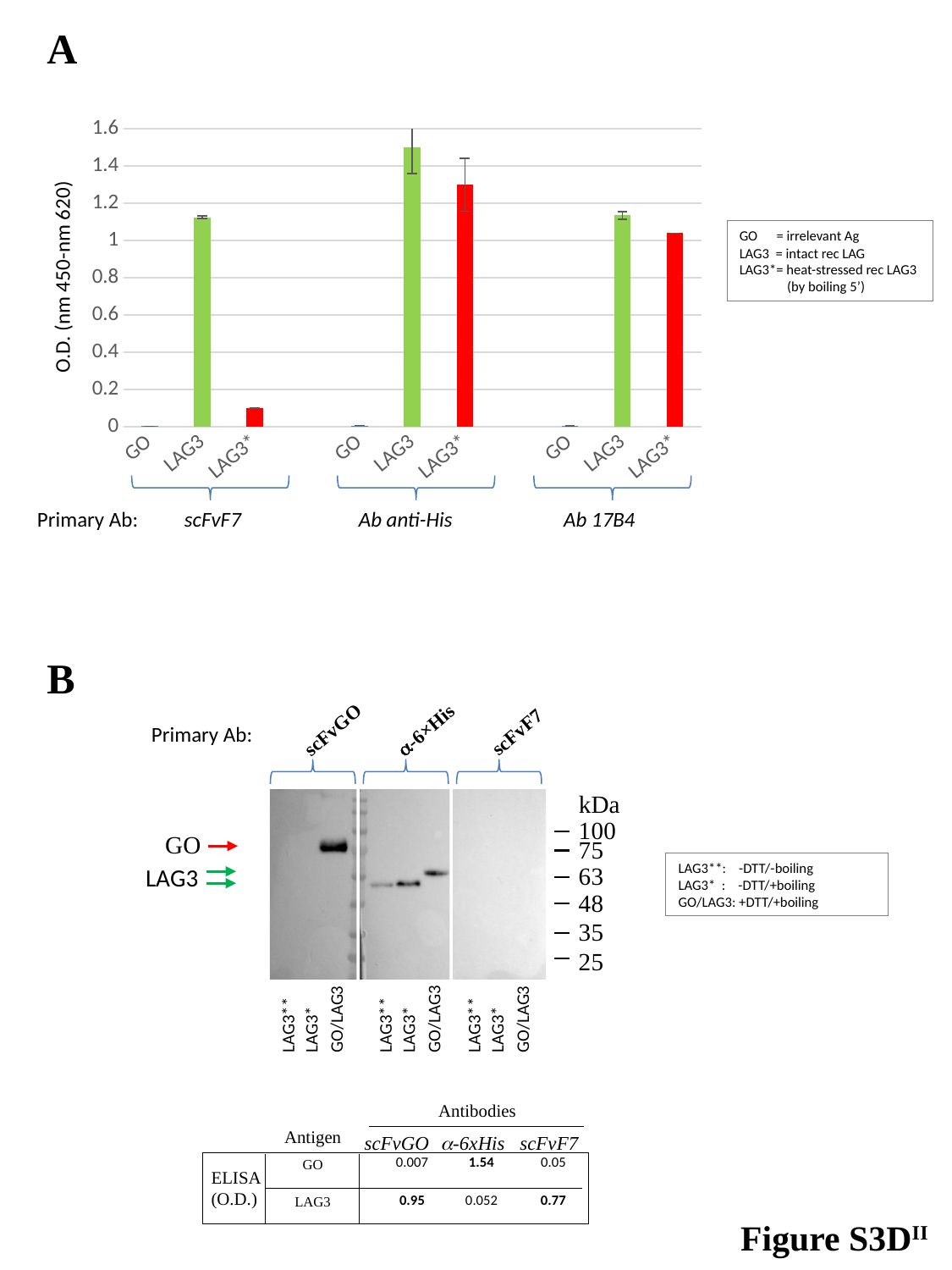

A
### Chart
| Category | |
|---|---|
| GO | 0.0035 |
| LAG3 | 1.125 |
| LAG3* | 0.1 |
| | None |
| GO | 0.007 |
| LAG3 | 1.5 |
| LAG3* | 1.2999999999999998 |
| | None |
| GO | 0.006 |
| LAG3 | 1.135 |
| LAG3* | 1.04 |O.D. (nm 450-nm 620)
scFvF7
 Ab anti-His
Ab 17B4
GO = irrelevant Ag
LAG3 = intact rec LAG
LAG3*= heat-stressed rec LAG3
 (by boiling 5’)
Primary Ab:
B
scFvGO
α-6×His
scFvF7
Primary Ab:
kDa
100
75
63
48
35
25
 GO
LAG3**: -DTT/-boiling
LAG3* : -DTT/+boiling
GO/LAG3: +DTT/+boiling
LAG3
LAG3**
LAG3*
GO/LAG3
LAG3**
LAG3*
GO/LAG3
LAG3**
LAG3*
GO/LAG3
Antibodies
Antigen
scFvGO
α-6xHis
scFvF7
| GO | 0.007 | 1.54 | 0.05 |
| --- | --- | --- | --- |
| LAG3 | 0.95 | 0.052 | 0.77 |
ELISA
(O.D.)
Figure S3DII
