# Supplementary material for: Development of a novel human phage display-derived anti-LAG3 scFv antibody targeting CD8+ T lymphocyte exhaustion
Source: BMC Biotechnol. 2019 Oct 17;19:67. doi: 10.1186/s12896-019-0559-x (PMC6798348; doi:10.1186/s12896-019-0559-x)
Supplement: Supplementary file 2 — Additional file 2: Figure S7AII. The treatment with the divalent scFvF7-Fc Ab increases the activation of peptide-stimulated Nef-specific CD8+ T lymphocytes in terms of IFN-γ secretion (Exp. I and Exp. III). For details see Legend of Fig. 7a. (PPTX 40 kb) [file 12896_2019_559_MOESM2_ESM.pptx]

## Slide 1
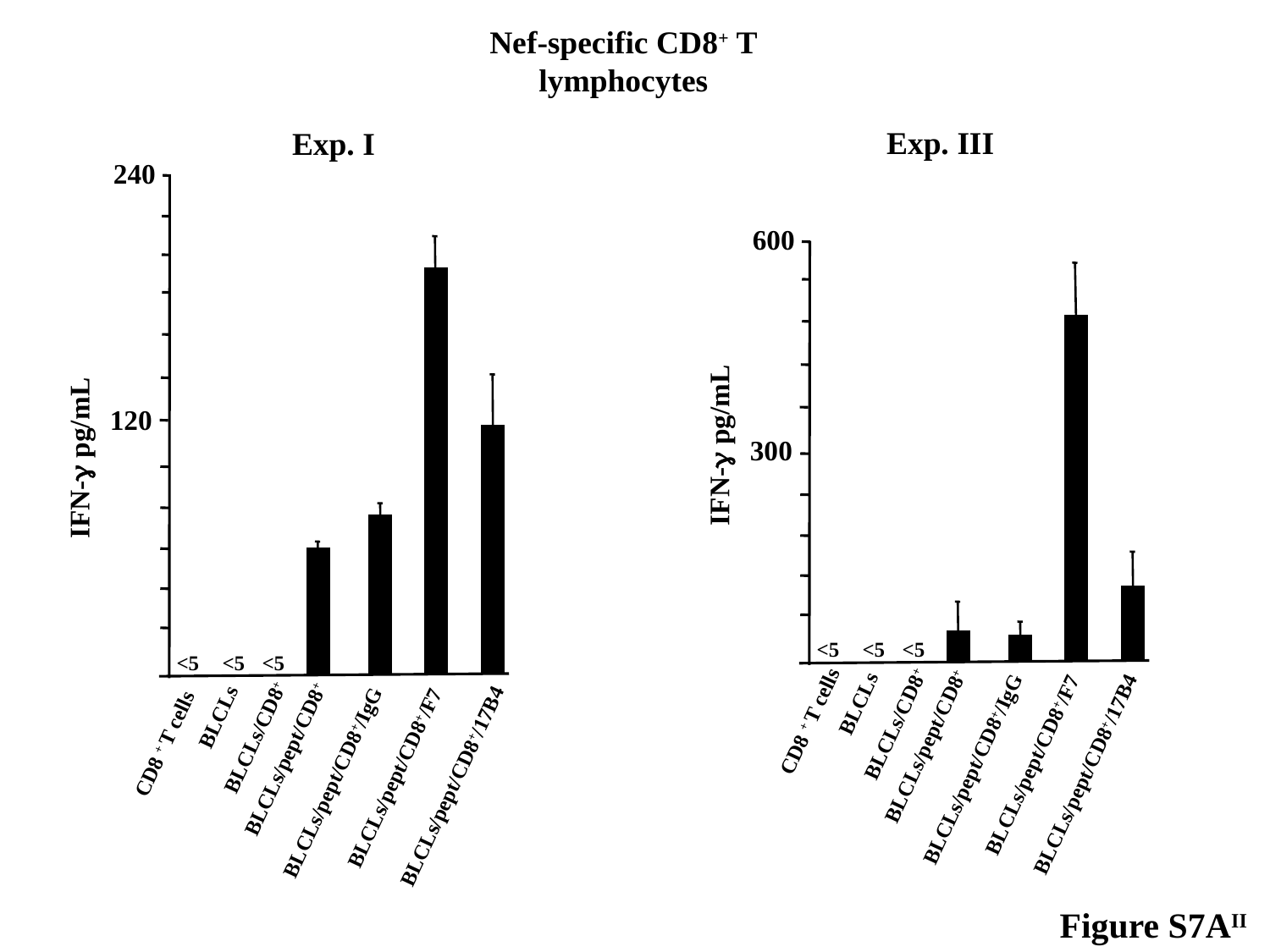

Nef-specific CD8+ T
lymphocytes
Exp. III
Exp. I
240
600
120
IFN-g pg/mL
300
IFN-g pg/mL
<5
<5
<5
<5
<5
<5
BLCLs
BLCLs
CD8 + T cells
BLCLs/CD8+
BLCLs/CD8+
CD8 + T cells
BLCLs/pept/CD8+
BLCLs/pept/CD8+
BLCLs/pept/CD8+/F7
BLCLs/pept/CD8+/IgG
BLCLs/pept/CD8+/17B4
BLCLs/pept/CD8+/F7
BLCLs/pept/CD8+/IgG
BLCLs/pept/CD8+/17B4
Figure S7AII
