# Supplementary figures and images for: Development of a novel human phage display-derived anti-LAG3 scFv antibody targeting CD8+ T lymphocyte exhaustion
Source: BMC Biotechnol. 2019 Oct 17;19:67. doi: 10.1186/s12896-019-0559-x (PMC6798348; doi:10.1186/s12896-019-0559-x)

## Slide 1
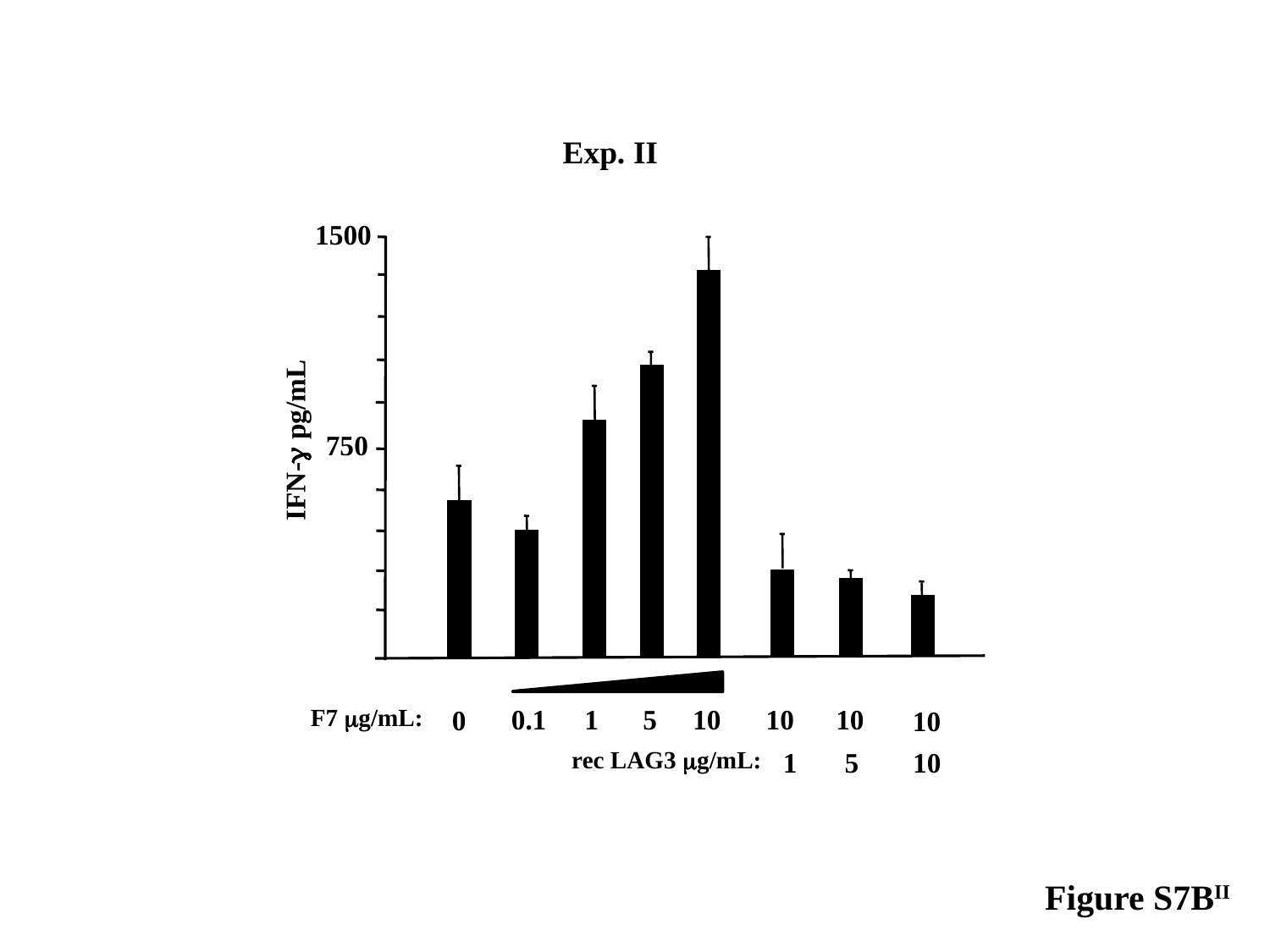

Exp. II
1500
IFN-g pg/mL
750
0.1
1
5
10
10
10
F7 mg/mL:
0
10
rec LAG3 mg/mL:
1
5
10
Figure S7BII

Supplement: Supplementary file 4 — Additional file 4: Figure S7BII. Dose-response effect of the divalent scFvF7-Fc Ab and inhibition by recombinant LAG3 (Exp. II). For details see Legend of Fig. 7b. (PPTX 36 kb) [file 12896_2019_559_MOESM4_ESM.pptx]
