# Supplementary material for: Development of a novel human phage display-derived anti-LAG3 scFv antibody targeting CD8+ T lymphocyte exhaustion
Source: BMC Biotechnol. 2019 Oct 17;19:67. doi: 10.1186/s12896-019-0559-x (PMC6798348; doi:10.1186/s12896-019-0559-x)
Supplement: Supplementary file 5 — Additional file 5: Figure S7CII. Effects of the divalent scFvF7-Fc as sensed by IFN-γ ELISPOT assay (Exp. II). For details see Legend of Figure 7c. (PPTX 36 kb) [file 12896_2019_559_MOESM5_ESM.pptx]

## Slide 1
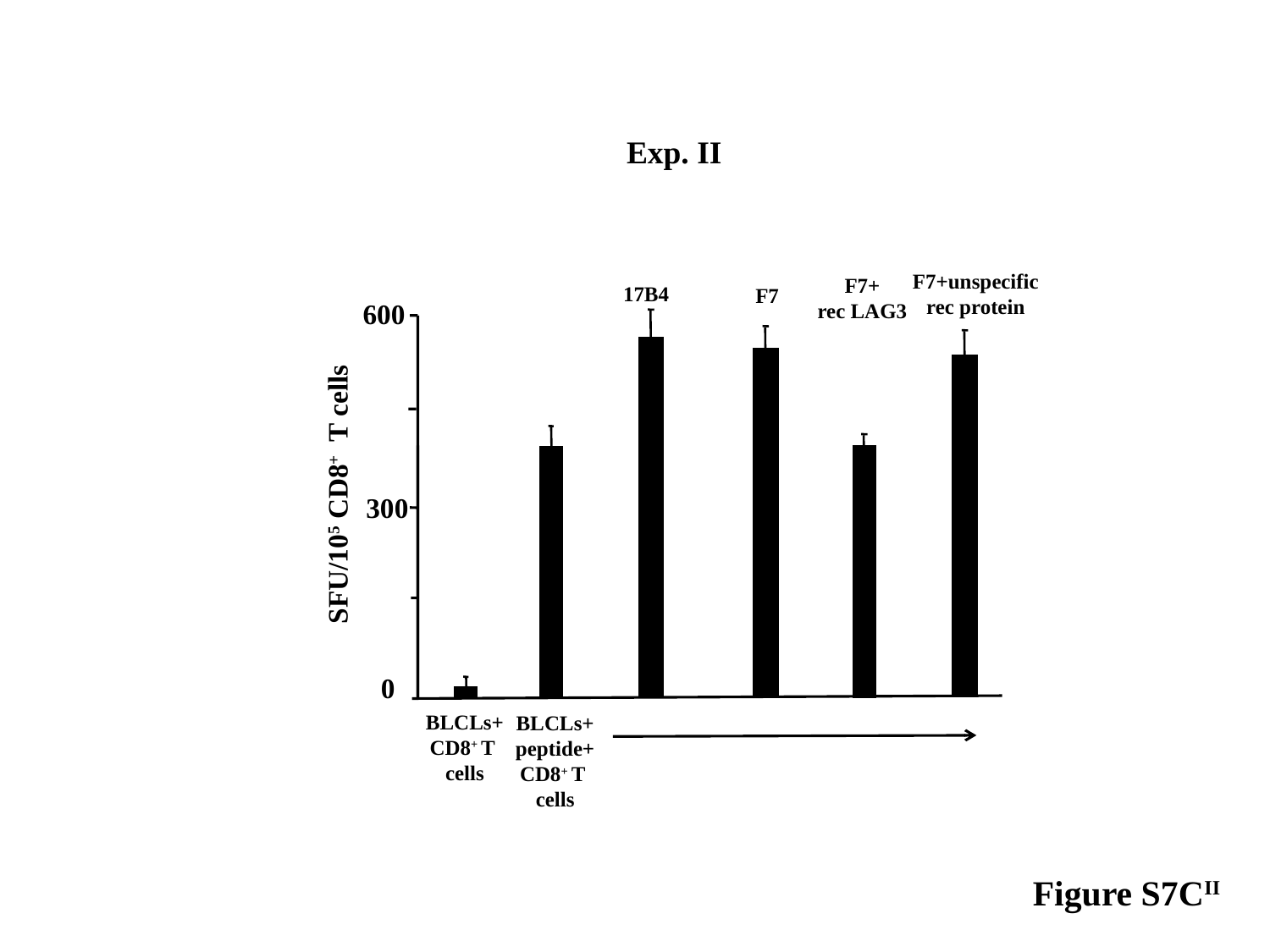

Exp. II
F7+unspecific
rec protein
F7+
rec LAG3
17B4
F7
600
SFU/105 CD8+ T cells
300
0
BLCLs+
CD8+ T
cells
BLCLs+
peptide+
CD8+ T
cells
Figure S7CII
